# 3D Printed Hydrogel Sensor for Rapid Colorimetric Detection of Salivary pH

**DOI:** 10.3390/s24123740

**Published:** 2024-06-08

**Authors:** Magdalena B. Łabowska, Agnieszka Krakos, Wojciech Kubicki

**Affiliations:** 1 Department of Mechanics, Materials and Biomedical Engineering, Faculty of Mechanical Engineering, Wroclaw University of Science and Technology, Smoluchowskiego 25, 50-371 Wroclaw, Poland; 2 Department of Microsystems, Faculty of Electronics, Photonics and Microsystems, Wroclaw University of Science and Technology, Janiszewskiego 11/17, 50-372 Wroclaw, Poland; agnieszka.krakos@pwr.edu.pl (A.K.); wojciech.kubicki@pwr.edu.pl (W.K.)

**Keywords:** pH sensor, hydrogel ink, bioplotting, 3D printing, hydrogel sensor, colorimetric detection, salivary diagnostics

## Abstract

Salivary pH is one of the crucial biomarkers used for non-invasive diagnosis of intraoral diseases, as well as general health conditions. However, standard pH sensors are usually too bulky, expensive, and impractical for routine use outside laboratory settings. Herein, a miniature hydrogel sensor, which enables quick and simple colorimetric detection of pH level, is shown. The sensor structure was manufactured from non-toxic hydrogel ink and patterned in the form of a matrix with 5 mm × 5 mm × 1 mm individual sensing pads using a 3D printing technique (bioplotting). The authors’ ink composition, which contains sodium alginate, polyvinylpyrrolidone, and bromothymol blue indicator, enables repeatable and stable color response to different pH levels. The developed analysis software with an easy-to-use graphical user interface extracts the R(ed), G(reen), and B(lue) components of the color image of the hydrogel pads, and evaluates the pH value in a second. A calibration curve used for the analysis was obtained in a pH range of 3.5 to 9.0 using a laboratory pH meter as a reference. Validation of the sensor was performed on samples of artificial saliva for medical use and its mixtures with beverages of different pH values (lemon juice, coffee, black and green tea, bottled and tap water), and correct responses to acidic and alkaline solutions were observed. The matrix of square sensing pads used in this study provided multiple parallel responses for parametric tests, but the applied 3D printing method and ink composition enable easy adjustment of the shape of the sensing layer to other desired patterns and sizes. Additional mechanical tests of the hydrogel layers confirmed the relatively high quality and durability of the sensor structure. The solution presented here, comprising 3D printed hydrogel sensor pads, simple colorimetric detection, and graphical software for signal processing, opens the way to development of miniature and biocompatible diagnostic devices in the form of flexible, wearable, or intraoral sensors for prospective application in personalized medicine and point-of-care diagnosis.

## 1. Introduction

The interest in biocompatible polymer materials has dynamically increased in the biomedical and biopharma sectors in the last few years [1,2,3]. Among them, hydrogels provide unique physiochemical properties, including relatively good mechanical stability, high liquid absorption, and insolubility, allowing versatile applications where biocompatibility and fabrication feasibility are critical [4,5]. Hydrogel layers have been commonly used as smart, pain-relieving wound patches [6,7], as well as precise structural scaffolds for cell culture and tissue engineering [8,9]. On the other hand, the responsive character of hydrogels, showing notable changes in their rheology, resistivity, or color tunability when exposed to variable external environments, make them a perfect tool for biosensing applications [10,11], including pH measurements.

Recent advances in additive manufacturing techniques have provided new possibilities for the fabrication of hydrogel-based devices [12,13]. Hydrogel inks of different content can be printed automatically, rapidly, and more easily in the process of bioplotting. This technique enables the formation of repeatable and more advanced hydrogel structures compared to manual methods [14,15]. As mentioned above, hydrogel matrices find application in cell culture, ensuring the 3D spheroid growth of microbials [16,17]. These microfluidic-based hydrogel platforms provide a passive physiological cellular environment and also become novel integrated perfusion systems, with drugs encapsulated in a polymer protective cover [18]. These approaches are interesting and commonly available alternatives to standard lab-on-chip technologies [19], which are based on more demanding and advanced silicon, glass, and PDMS microengineering.

Bioplotting of hydrogel matrices also opens the way for dedicated, low-cost, and biodegradable sensor platforms. According to the state of the art [20,21,22], considerable attention has been paid to hydrogel biosensors and their possible role as new diagnostic tools for modern and personalized medicine. In this regard, hydrogel matrices can be used as stimuli-responsive materials, as immobilization substrates for sensing molecules, and as protection surfaces. However, the main focus has recently been on the development of simple and non-toxic biosensors that allow the real-time monitoring of chemical biomarkers in human fluids in a wide detection range [23,24,25]. Such point-of-care devices can be used in a disposable way, for single ex vivo use, and in a constant wearable mode. As much effort has recently been put to provide non-invasive medical diagnostics, the monitoring of potential health disfunctions based on tears, sweat, or saliva can provide a real revolution in biopharma strategies.

Saliva, also called a “mirror of the body”, is a valuable diagnostic specimen that can directly reflect many pathological states of the human body condition [26]. Due to the ease of sample collection, storage, and acceptable sensitivity, saliva evaluation can constitute an important alternative to standard blood examination, which without the need for qualified personnel appears to be a perfect solution for home care devices [27]. Variations in saliva pH levels can be an indication of a number of health issues, including systemic illnesses, metabolic disorders, and malfunctioning salivary glands. For instance, too high or too low saliva pH can be a sign of various health issues, whereas too low pH can encourage gum disease and tooth decay. Salivary diagnostics may provide rapid insight into both intraoral disfunctions (e.g., periodontal diseases), as well as serious systemic disorders (e.g., diabetes mellitus, cancer). According to the most current WHO guides, intraoral testing can be a chance for developing countries to provide inexpensive and easy-to-use medical diagnostics, notably improving the rapid profiling of health conditions in underserved groups [28,29]. However, the general need for accurate and low-cost biosensors, produced using available and simple fabrication methods, is a key issue that requires global interest and novel development strategies.

A recent trend toward the use of biocompatible hydrogel matrices that are sensitive to local environmental changes (e.g., pH, temperature, gases) and the presence of specific biomarkers (e.g., glucose, urea, antigens, drugs) in combination with microfluidics and microsystem techniques can provide new solutions of both extraoral and intraoral biosensors. Accompanied by rapid bioplotting technology, a new generation of portable salivary diagnostic systems can be developed. Thanks to the unique properties of hydrogels, different sensing methods are available, strictly related to their composition and application. The most popular are hydrogel sensors based on colorimetry, conductance, and piezoresistivity. Utilizing hydrogel sensors for determining changes in pH in saliva is useful to assess the condition of the oral mucosa and detect metabolic disorders, dysfunctional salivary glands, candida, and even systemic diseases like diabetes, gastroesophageal reflux, cancer, or autoimmune diseases [30].

For example, Wen et al. [31] proposed a polyacrylamide structure for the detection of ammonia based on the color change of the hydrogel induced by the pH value. A similar approach was presented in the work of Tang et al. [32], where a composition of polyvinyl alcohol and sodium alginate was used as a functional layer of the hydrogel. Lee et al. [33] and Wu et al. [34] also presented an interesting solution to enable the rapid detection of glucose using agarose and methacrylate gelatin (GelMA). Apart from the color change of hydrogels exposed to water solution, including different proportions of glucose, a visible shrinkage in the hydrogel geometry was observed in such structures [34].

Conductance detection, as well as piezoresistivity changes, are in turn typically used for ethanol [35,36] and drug detection [37]. The literature review shows that the hydrogels based on gelatin, polyacrylamide, and bisacrylamide are the most common in this regard. Nevertheless, in these cases, the hydrogel sensor fabrication procedures may seem rather complicated, often requiring multistep protocols as well as specialized equipment and laboratories [25,38]. Moreover, in the case of exclusively bisacrylamide structures, these solutions are considered toxic [39], thus the potential application of such sensors for near-oral diagnostics is rather controversial.

Based on the aforementioned issues, the preparation of a hydrogel sensor with total biocompatibility and simplified manufacturing steps is not trivial and needs further investigation. In this work, a hydrogel structure for pH assessment toward salivary diagnostics utilizing rapid colorimetry detection is shown. Non-toxic and polymer inks of unique but simple and available composition were prepared, based on sodium alginate and polyvinylpyrrolidone, for simplified and automated bioplotting of the small-scale sensor matrices. Dedicated software was developed to quickly and easily process and analyze the color tunability of the hydrogel sensor exposed to pH changes. The hydrogel structures proposed in this work were investigated in terms of mechanical properties (tensile tests) and sensing capabilities in response to defined saliva samples (raw or with additives). The results of the experiments confirmed fine mechanical durability and appropriate operational stability of the sensor, as well as quick response to pH changes, which in combination with simple software-based signal detection reveal the prospective application of the device in future point-of-care diagnostics.

## 2. Materials and Methods

### 2.1. Fabrication of the Hydrogel pH Sensor

**Figure 1:**
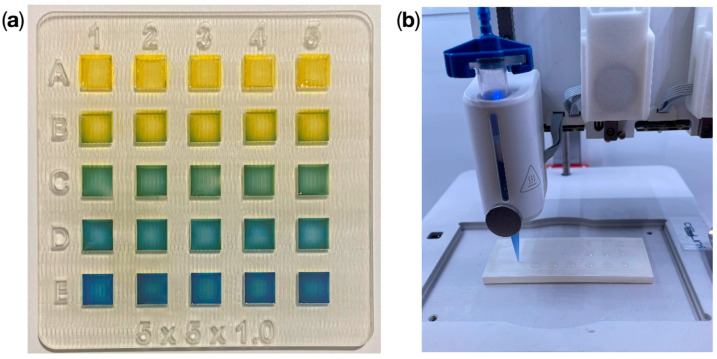
Preparation of the hydrogel pH sensors: (**a**) 3D printed substrate (outer dimensions: 54 × 51 × 1.1 mm^3^) containing a matrix of 25 bioplotted 5 × 5 mm^2^ hydrogel sensor pads, (**b**) hydrogel pattern bioplotting process utilizing a BioX Cellink printer.

Hydrogel pads were bioplotted on a dedicated substrate, which contained a matrix of 25 square cavities, 5 × 5 × 1 mm^3^ each (Figure 1a). The matrix was used to enable easy and repeatable capture of the image for testing various pH samples at the same conditions. However, the sensing hydrogel layer may be plotted onto other surfaces and in a more sophisticated pattern on demand. The substrate was manufactured using a multi-jet 3D printing technique (Projet 3500 SD Max, 3D Systems, Rock Hill, SC, USA) using photocurable and biodegradable VisiJet M3 Crystal construction material (3D Systems, Rock Hill, SC, USA) and VisiJet S300 support material (3D Systems, Rock Hill, SC, USA). The substrate was treated to obtain repeatable surface properties, according to the post-processing protocol described elsewhere [40]. Post-processing included an air heating process followed by an oil bath at 65 °C, a water-based detergent bath in an ultrasonic cleaner, and a final washing step with isopropyl alcohol (IPA).

The hydrogel ink solution was composed of 5% (*v*/*v*) sodium alginate (Sigma Aldrich, Saint Louis, MO, USA), 1% (*v*/*v*) polyvinylpyrrolidone (Sigma Aldrich, Saint Louis, MO, USA), and 0.1% (*v*/*v*) bromothymol blue pH indicator (Warchem, Zakręt, Poland). The chemical characteristics and their impact on the output hydrogel structure were taken into consideration during the choice of ink composition and the proportions of the ingredients. Physical characteristics, including the sensor material’s strength, permeability, absorption capacity, and stability under various environmental conditions, were also taken into account. The water-based mixture was prepared by stirring (400 RPM) at 60 °C for 60 min using a laboratory hot plate with a magnetic stirrer. Sensor pads were filled with a repeatable dose of a hydrogel ink utilizing a commercially available 3D printer (BioX, Cellink, San Diego, CA, USA) with the process parameters as follows: *p* = 20 kPa, v = 4 mm/s, and T = 30 °C (Figure 1b). Next, a 4% (*v*/*v*) calcium chloride solution (Warchem, Zakręt, Poland) was gently pipetted into the pads to enable cross-linking of the hydrogel structures in 10 min.

### 2.2. Mechanical Properties of the Hydrogel Structures

**Figure 2:**
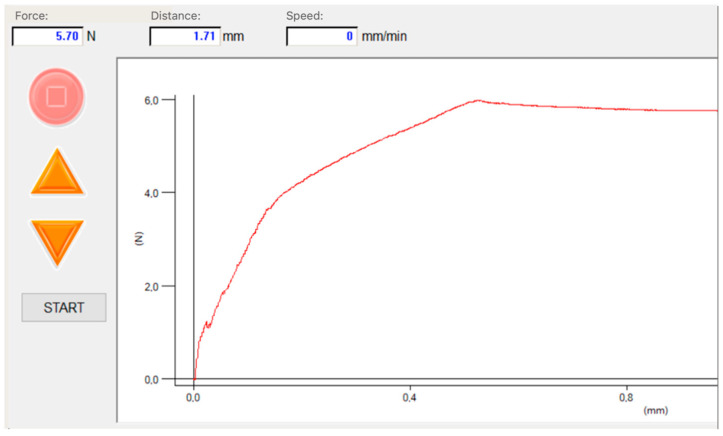
Tensile strength of the hydrogel matrices. Emperor Force software v1.18-408 for visualization of the maximum tensile strength.

Due to the high water content in their structure, hydrogels pose relatively poor mechanical properties compared to other materials. The mechanical durability of hydrogel materials depends mainly on the fluid content, as well as the strength of the bonds between the polymer chains of the structure, which are directly affected by the cross-linking process. Appropriate durability is essential to ensure the resilience of thin layers, preventing them from tearing during use and enabling prolonged storage capability. Furthermore, the use of additive manufacturing technology ensures precise deposition of layers of equal thickness, ensuring repeatable results. To assess the mechanical durability of the hydrogel layers, the tensile strength and compression tests were performed using the MultiTest-i-1 instrument (Mecmesin, Slinfold, United Kingdom). The apparatus was equipped with the ILC-S strain gauge sensor with a measuring range of 100 N, operating at a constant velocity of 5 mm/min (Figure 2). Tensile tests were conducted for both pure hydrogel structures (including sodium alginate and polyvinylpyrrolidone), as well as structures containing the pH indicator (bromothymol blue). Cuboidal hydrogel samples with dimensions 5 × 0.7 × 50 mm^3^ were fabricated on a 3D BioX bioplotter to ensure replicability. Mechanical tests were also conducted for hydrogel structures subjected to various pH environments to verify their potential shrinkage or swelling under different ambient conditions. In this case, a compression test was conducted on 5 × 5 × 5 mm^3^ hydrogel cuboids with a pH indicator included in the structures, placed in different pH solutions for 10 min. Three pH values (extremes and typical for saliva: pH 3.5, 9.0, and 6.5) were used for examination. Based on instrument force indication, Young’s modulus (E) and compressive modulus of elasticity (E_c_) values for the hydrogel samples were calculated.

Afterward, statistical analysis was performed with the Statistica package (version 13, TIBCO Software Inc., Palo Alto, CA, USA) to visualize the maximum, mean, and minimum values of Young’s modulus and the compressive modulus of elasticity for each group of samples in box-and-whisker chart form.

### 2.3. Colorimetric Detection—Software-Enhanced Analysis of the Sensor Responses

**Figure 3:**
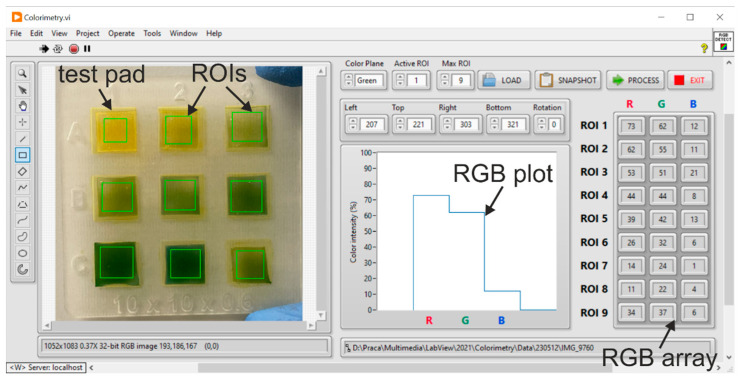
Front panel of the developed LabVIEW application for image colorimetry. RGB plots are automatically processed for selected rectangular ROIs selected in the image of the sensor matrix.

The response of the hydrogel sensor was obtained by first capturing a color image of the matrix using a 24 MP CMOS camera. Next, the image was digitally processed by dedicated software with a graphical user interface (LabVIEW, National Instruments), developed based on our previous experience with optical signal processing [41,42]. As the color of the individual pad changed proportionally to the change of the pH level, the software-based algorithm was used to extract red (R), green (G), and blue (B) components from individual regions of interest (ROIs) of the image. The software enables manual or programmed selection of ROIs, and the measured data is presented in both graphical and tabularized forms (Figure 3). The numerical components of RGB plots are stored and may be compared, enabling measurement of pH values of the samples, as well as preparation of the calibration curve for the pH pattern (e.g., when the composition of the ink is changed). Due to the applied algorithm, the colorimetric detection is rapid and simple and can be performed for numerous pads in parallel. The shape of the ROIs can also be adapted to other patterns of the sensor pads.

### 2.4. Calibration Curve for Hue Values and Tests of the Hydrogel Sensor

All experiments were carried out under stabilized laboratory conditions at 22 °C and 35% humidity. The sensor calibration curve was obtained for a pH range of 3.5–9.0 with a 0.5 step. The calibration curve was determined based on the hue value, which is derived by the ratio of the dominant wavelength to other wavelengths in the color, indicated by its position (in degrees) on the RGB color wheel: H = [0°, 360°]. The hue value can be calculated based on the RGB components from Formula (1).

(1) (A) H=60°×[(G′−B′)/(R′−B′)]  for R ≥ G ≥ B

(B) H=60°×[2−(R′−B′)/(G′−B′)] for G> R ≥ B

(C)H=60°×[2+(B′−R′)/(G′−R′)] for G ≥ B> R

(D) H=60°×[4−(G′−R′)/(B′−R′)] for B> G> R

(E) H=60°×[4+(R′−G′)/(B′−G′)] for B> R ≥ G

(F) H=60°×[6−(B′−G′)/(R′−G′)] for R ≥ B> G

where R’G’B’ value are calculated from:

R′=(R value)/255G′=(G value)/255B′=(B value)/255

Next, a ready-to-use hydrogel sensor was tested with solutions of artificial saliva for medical and dental research (Pickering Labs, Mountain View, CA, USA) and its mixtures with beverages: lemon juice, black tea, green tea, bottled water, tap water, and coffee. The concentrations of the beverages examined included 100% (pure beverage) and their mixtures with artificial saliva in proportions of 50%, 20%, and 10%. Furthermore, the examination involved sensor response time (pH-based color change) as a function of time. Therefore, colorimetric data were collected every 30 s within the first 5 min after the application of the test sample, and additionally after 10 and 15 min.

## 3. Results and Discussion

### 3.1. Mechanical Properties of the Bioplotted Hydrogel Matrices

**Figure 4:**
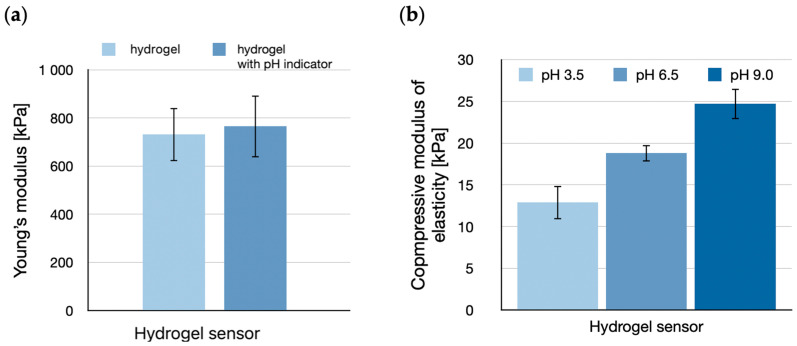
Tensile strength tests of the hydrogel sensor. (**a**) Young’s modulus (E) of hydrogel and hydrogel with pH indicator added (N = 10), (**b**) comparison of the compressive modulus of elasticity (E_c_) of hydrogel exposed to different pH (3.5, 6.5, 9.0) (N = 10).

Mechanical strength tests of hydrogel structures based on sodium alginate and polyvinylpyrrolidone showed a Young’s modulus (E) value of 731 ± 110 kPa, whereas the addition of the bromothymol blue pH indicator slightly increased the value to 765 ± 128 kPa (Figure 4a). Based on the change in pH of the environment, sodium alginate-based hydrogels react by shrinking or swelling, which directly affects the strength of the polymer’s chemical bonds. This relationship may affect the degree of response of the pH indicator incorporated into the hydrogel structure. Compressive modulus of elasticity (E_C_) values increase with pH increases, therefore E_C_ for pH 3.5 is 12.9 ± 2 kPa, for pH 6.5 is 18.8 ± 1 kPa, and for pH 9.0 is 24.7 ± 1.8 kPa (Figure 4b).

### 3.2. Calibration of the Colorimetric Hydrogel Sensor and pH Salivary Diagnostics

**Figure 5:**
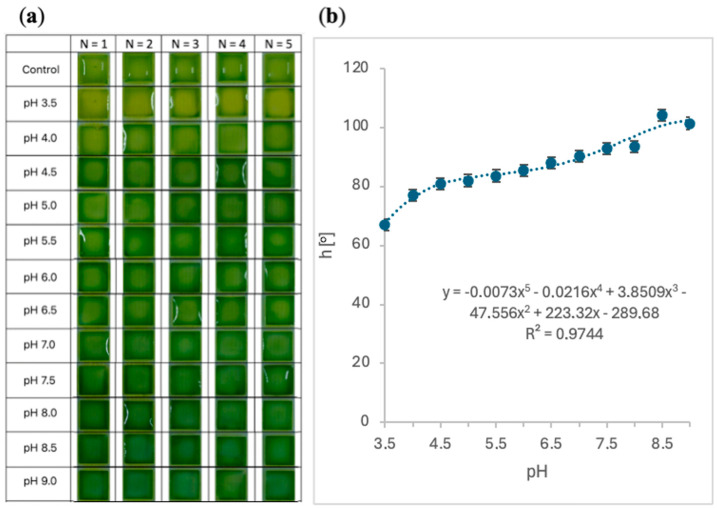
Colorimetry response of hydrogel sensor to pH values in the range 3.5 to 9.0. (**a**) Visible color tunability of hydrogel sensors after 30 s of various pH application, (**b**) calibration curve for hue values as a function of pH with fitted polynomial curve after 30 s of various pH application (mean values, N = 5).

In Figure 5a, the sensor calibration curve is presented. As shown, hydrogel pads containing bromothymol blue pH indicator provided appropriate responses to the defined pH solutions. Ranging from 3.5 to 9.0 with 0.5 step, visible color tunability of the hydrogel pads can be observed 30 s after measurement begins. Observation by the naked eye suggests the color change from light green for acidic solutions to dark green for alkaline solutions (Figure 5a), but the RGB analysis clearly indicated an increase in the sum of the green and red components (resulting in a yellow hue) in the first case and a blue component in the latter, which confirms the proper response of the bromothymol blue indicator [43]. Moreover, software-enhanced analysis of the responses of the sensors was conducted and presented in Figure 5b in the form of the hue values as a function of pH values. The tendency of the components R and G to decrease with the simultaneous increase of the B component is consistent with the decimal code tables [44], in which R and G achieved maximum values for the yellow color, and minimum for the blue one. These results confirmed the appropriate operation of the hydrogel sensor and suggested further research with artificial salivary samples.

**Figure 6:**
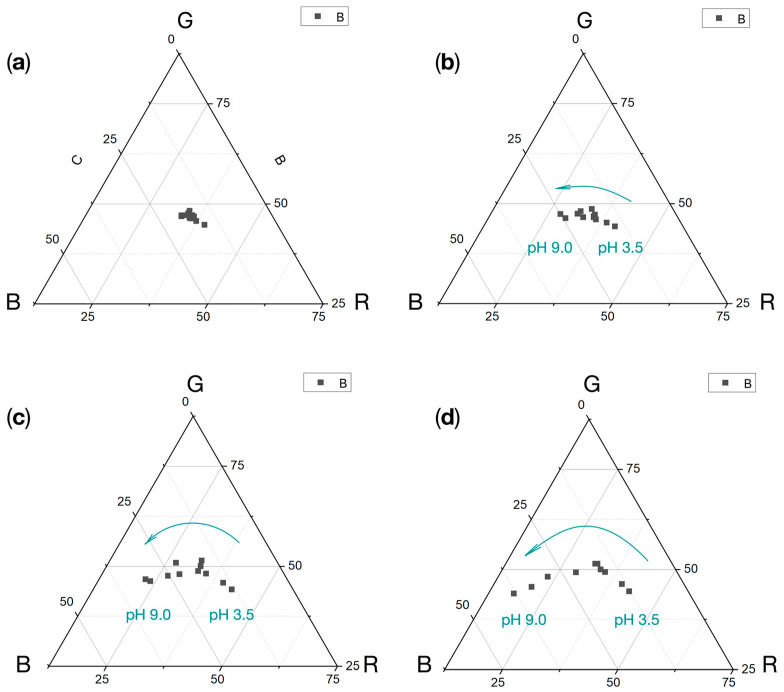
Triangle charts of RGB components for color changes for various pH 3.5–9.0 (mean values, N = 5) at (**a**) 0 min, (**b**) 1 min, (**c**) 5 min, (**d**) 15 min.

The investigation shows repeatability of measurements, where this is noticeable in the triangular plot for the hydrogel sensor control group (Figure 6a). The sensor shows an increase in the values of the color components in the time function, slight for acidic solutions (pH 3.5, Figure 6b) and noticeable for neutral solutions (pH 7.0, Figure 6c) and alkaline solutions (pH 9.0, Figure 6d). An acidic reaction causes shifts toward the R component, for neutrals toward the G component, and for alkalis toward the B component.

**Figure 7:**
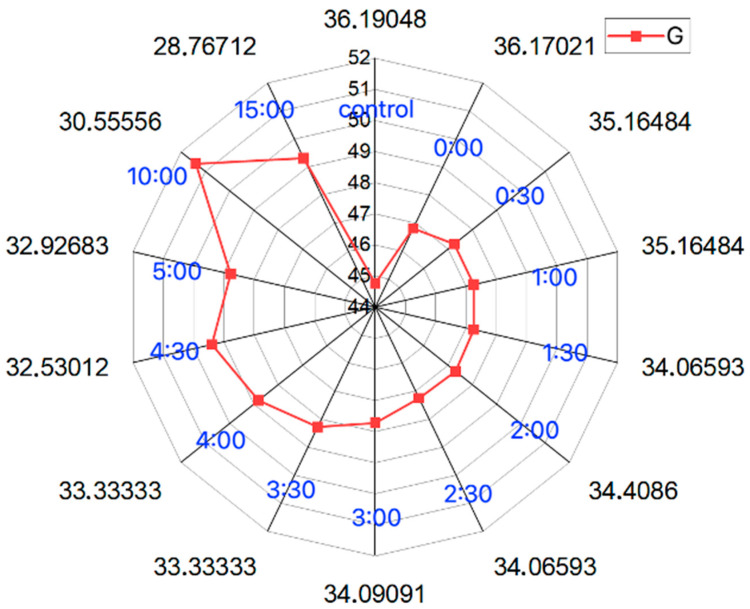
Radar chart of hydrogel sensor responses at 0–15 min (example for ph 6.5 solution)—stability of the hydrogel sensor (mean values, N = 5).

The stability of the sensor is shown using a radar plot (Figure 7), where similar results can be seen in the analysis of the RGB components from the beginning of the measurement to 4 min with a slight upward trend toward the G component and a slight downward trend toward R component. Measurements after 4 min show larger changes in component values, which may be the result of a chemical reaction of the indicator or evaporation of water from the hydrogel structure of the sensor.

### 3.3. Measure of Salivary pH and pH of Saliva Mixed with Beverages

**Figure 8:**
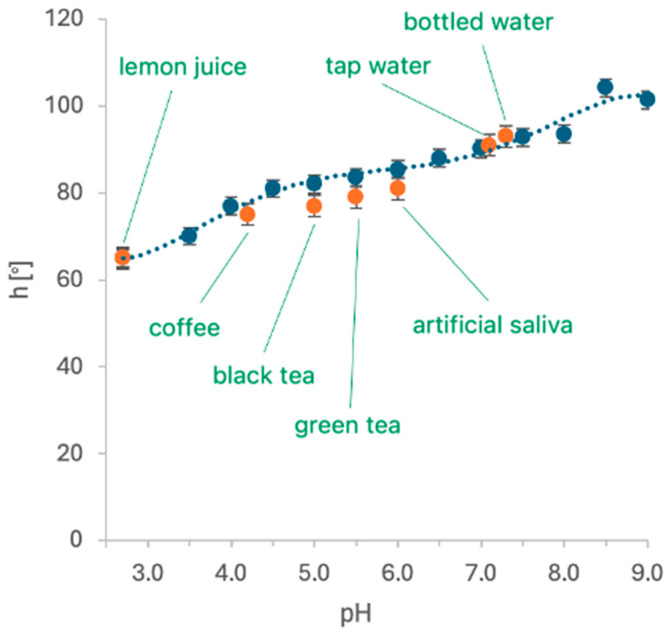
Hue values of beverages as a function of pH after 30 s of measurement (mean values, N = 5).

**Figure 9:**
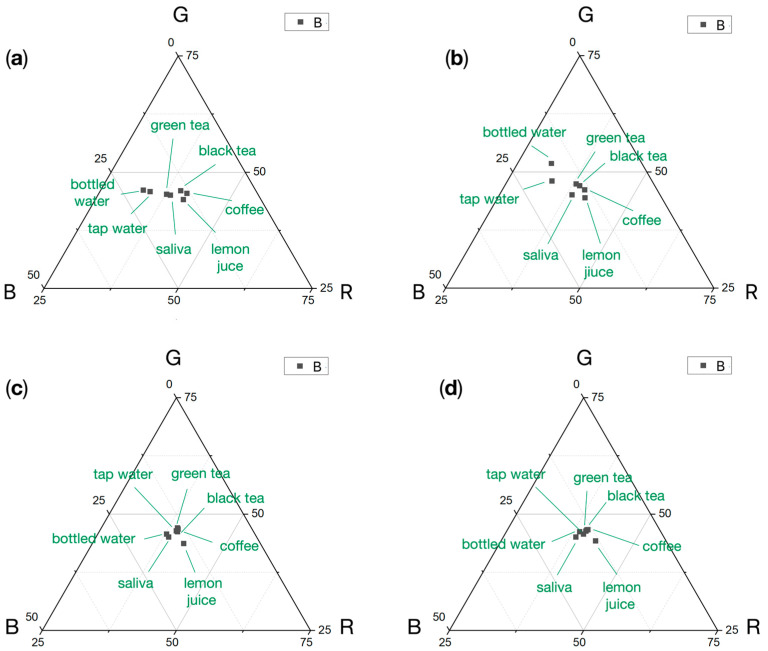
Triangle chart of the RGB component for hydrogel sensor responses to saliva and saliva-beverages mixtures after 15 min of application (mean values, N = 5); (**a**) beverages, 100% concentration; (**b**) beverages and saliva mixtures, 50% concentration; (**c**) beverages and saliva mixtures, 20% concentration; (**d**) beverages and saliva mixtures, 10% concentration.

In this paper, the hydrogel sensor response to color changes under the influence of the pH of synthetic saliva, beverages (100%), and a mixture of saliva and beverages in ratios of 50%, 20%, and 10% were examined. Prior to the experiments, artificial saliva and saliva with the beverage mixture were measured with a pH meter. The pH values for each beverage were as follows: artificial saliva—pH 6.0, lemon juice—pH 2.3, coffee—pH 4.2, black tea—5.0, green tea—pH 5.5, tap water—7.1, bottled water—pH 7.3. The pH measurements of each beverage for hue values were plotted onto the calibration curve (Figure 8). At the same time, the pH values of the artificial saliva and the beverage mixture changed due to the pH value of the saliva. The values of the RGB components of the color-changing hydrogel under the influence of a mixture of beverages and saliva are shown in triangular charts in Figure 9 for 50% concentration.

Analysis of RGB components shows noticeable changes in the presence of beverages in saliva at a concentration of 50%. Minor correlations were observed for the concentration of 20% and 10% beverage content in saliva. While more acidic reactions of the substances tested tended to shift toward the R and G components (successively, mixtures with lemon juice, coffee, black tea, green tea, and pure artificial saliva), for the neutral reaction, a trend of increase toward the B component was observed (values close to pH 7 for bottled water and tap water).

## 4. Conclusions

The research carried out in this study showed the possibility of fabrication using additive manufacturing technologies of a colorimetric hydrogel sensor based on sodium alginate and polyvinylpyrrolidone with the addition of a pH indicator. The results showed the possibility of a fast (30 s) and reproducible response to pH range (3.5 to 9.0) with simultaneous high sensor stability. To confirm the durability of the bioplotted hydrogel sensor, the mechanical properties of the sensor were tested, which showed the strength of the thin films, reaching above 700 kPa of Young’s modulus value. For the purpose of the sensor validation, dedicated software with an easy-to-use graphical user interface was developed to extract the R(ed), G(reen), and B(lue) components of the color image of the hydrogel pads and evaluate the pH value. After a sensor calibration, it was validated using samples of artificial saliva and its mixtures with beverages (lemon juice, coffee, black and green tea, bottled and tap water). As a result, a clear response to acidic and alkaline solutions of the sensor was observed, revealed in the form of substantial color change, visible also with the naked eye. However, consistent with the recent literature reports, the influence of different reactions on the hydrogel structure can lead to shrinkage of the hydrogel (in acidic conditions) or swelling of the hydrogel (in neutral and alkaline environments). This can, in turn, affect the rate of response, relax the polymer chains upon swelling, and release the pH indicator from the material structure. On that basis, further research to enhance the sensor reliability and provide the best pH indications is needed. Attention should also be paid to the rate of evaporation of water from the hydrogel structure, and methods should be developed to enable the preservation or storage of the sensor after its production to prevent the hydrogel from drying out.

## Data Availability

The raw data supporting the conclusions of this article will be made available by the authors on request.
